# Cytokines at the Interplay Between Asthma and Atherosclerosis?

**DOI:** 10.3389/fphar.2020.00166

**Published:** 2020-03-04

**Authors:** Danila Gurgone, Lucy McShane, Charles McSharry, Tomasz J. Guzik, Pasquale Maffia

**Affiliations:** ^1^ Centre for Immunobiology, Institute of Infection, Immunity and Inflammation, College of Medical, Veterinary and Life Sciences, University of Glasgow, Glasgow, United Kingdom; ^2^ Department of Pharmacy, University of Naples Federico II, Naples, Italy; ^3^ Institute of Cardiovascular and Medical Sciences, College of Medical, Veterinary and Life Sciences, University of Glasgow, Glasgow, United Kingdom; ^4^ Department of Internal and Agricultural Medicine, Jagiellonian University College of Medicine, Kraków, Poland

**Keywords:** asthma, atherosclerosis, cardiovascular disease, cytokines, interleukin

## Abstract

Cardiovascular disease (CVD) is an important comorbidity in a number of chronic inflammatory diseases. However, evidence in highly prevalent respiratory disease such as asthma are still limited. Epidemiological and clinical data are not univocal in supporting the hypothesis that asthma and CVD are linked and the mechanisms of this relationship remain poorly defined. In this review, we explore the relationship between asthma and cardiovascular disease, with a specific focus on cytokine contribution to vascular dysfunction and atherosclerosis. This is important in the context of recent evidence linking broad inflammatory signaling to cardiovascular events. However inflammatory regulation in asthma is different to the one typically observed in atherosclerosis. We focus on the contribution of cytokine networks encompassing IL-4, IL-6, IL-9, IL-17A, IL-33 but also IFN-γ and TNF-α to vascular dysfunction in atherosclerosis. In doing so we highlight areas of unmet need and possible therapeutic implications.

## Introduction

Cardiovascular disease (CVD) is the underlying cause of around 30% of deaths worldwide, approximately 80% of which stem from the underlying pathology of atherosclerosis causing the clinical manifestations of myocardial infarction (MI) and ischemic stroke (WHO, 2011). It is well established that inflammation and the immune system contribute to the development of atherosclerosis, along with various other cardiovascular disorders (Harrison et al., 2011; Hu et al., 2015; Welsh et al., 2017; MacRitchie et al., 2020). As a result, clinical research is now progressing in this area pioneered by the CANTOS (Canakinumab Anti-Inflammatory Thrombosis Outcomes Study) trial, the results of which were released in the last 2 years (Ridker et al., 2017). This trial found that pharmacological inhibition of cytokine interleukin-1β (IL-1β) with the monoclonal antibody Canakinumab significantly reduced subsequent cardiovascular events in people who had previously suffered a MI. Furthermore, patients which responded best to this treatment were those which had higher levels of circulating IL-6 and C-Reactive Protein (CRP), indicative of systemic inflammation (Maffia and Guzik, 2019). CANTOS trial has been followed by the CIRT trial (Cardiovascular Inflammation Reduction Trial), where treatment with low-dose methotrexate failed to lower cardiovascular event rates in patients with previous multivessel coronary artery disease or MI and affected by metabolic syndrome or type 2 diabetes (Ridker et al., 2019). These results confirmed that selective targeting of proinflammatory cytokines in atherosclerosis could be a beneficial addition to current treatments, which is dominated by cholesterol lowering drugs.

In light of the important role immune-inflammatory responses play in driving atherosclerosis it is unsurprising that patients suffering from chronic inflammatory diseases are more prone to CVD (Tseng et al., 2015; Liu C. et al., 2016; Czesnikiewicz-Guzik et al., 2019; Ferguson et al., 2019). However, the relationship between the highly prevalent disorder asthma, reported to affect around 10–25% of the population of Europe and more than 200 million people world-wide (https://ginasthma.org/), and CVD still needs to be fully elucidated. As such, further research in the area is strongly required, given that asthmatic patients may represent a large group of people at greater risk for developing CVD, a clinical burden which is not currently addressed (To et al., 2012; Strand et al., 2018).

**Figure 1:**
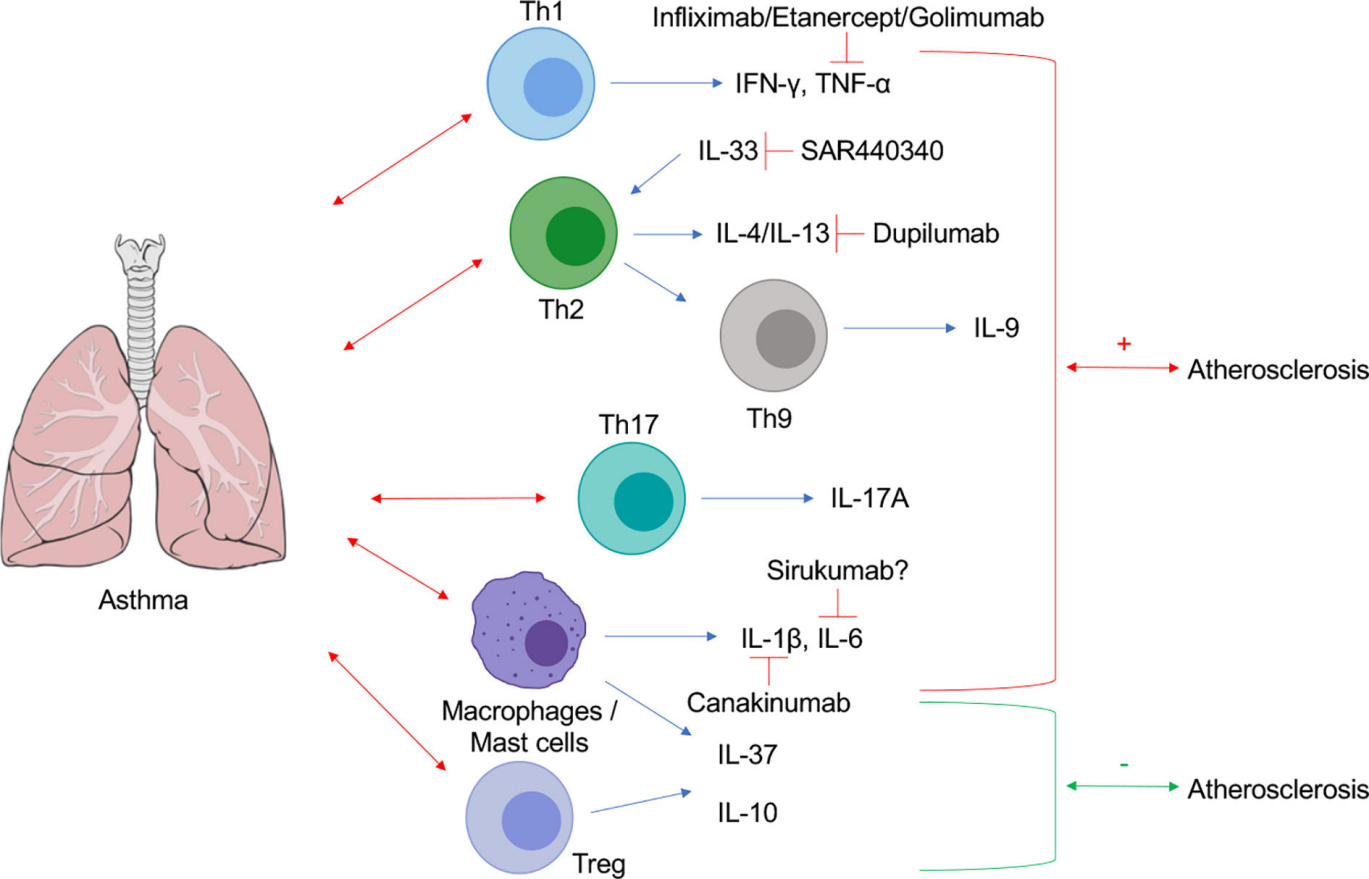
Schematic representation of cytokine contributions at the interplay between asthma and atherosclerosis. Lung Image Credit: Patrick J. Lynch, medical illustrator (Creative Commons Attribution CC BY 2.5) *via* Wikipedia.

The release of cytokines is central to almost every stage of the immune response in asthma, and consequent systemic dysregulation of inflammatory homeostasis may explain their potential higher risk of developing CVD. It may be plausible that targeting cytokines which are both highly expressed in asthma and causative in cardiovascular pathology may represent a pharmacological strategy for the treatment of accelerated CVD in asthma. Therefore, in this mini review, we will explore CVD comorbidity in asthma, and we will discuss whether cytokines may be potential pharmacological targets at the interface of these two disorders (**Figure 1**).

## Asthma Pathology and Cardiovascular Comorbidity

Asthma is a heterogeneous disease widely understood to be associated with airway hyperresponsiveness and inflammation. Pathological obstruction due to mucus production and structural changes in the airways, due to epithelium thickening and abnormal accumulation of airway smooth muscle cells, reduces airways caliber to generate the characteristic asthmatic symptoms (shortness of breath, coughing or wheezing, chest tightness or pain) (Holgate et al., 1999). The best known phenotype of asthma is allergic asthma, in which these symptoms are caused by inhaled allergens such as house dust mites, animal dander, fungi, and pollens (Kudo et al., 2013), triggering an immunological response driven by T helper type 2 (Th2) cells and their associated cytokines IL-4, IL-5, and IL-13 (Lloyd and Hessel, 2010). However, alternative inflammatory and non-inflammatory mechanisms for asthma have been described and it is now accepted that there are multiple phenotypes and endotypes of the pathology (Bossley et al., 2012; Fahy, 2015). Whether these distinctions confer susceptibility or protection from developing CVD remains unclear.

Asthma and atherosclerosis are both characterized by accumulation of immune cells at the site of injury, increased tissue and circulating levels of IgE, and activation of mast cells and smooth muscle cells (Willems et al., 2013; Liu C. et al., 2016). Asthma is also characterized by an elevation of circulating proinflammatory and Th2 cytokines, indicating that the vasculature is systemically exposed to the inflammation generated in the lungs (Wong et al., 2001; Huang et al., 2016; Zhu et al., 2016). Therefore, patients with asthma are exposed to an inflammatory environment that may favor atherosclerosis progression, even though, clinical data are not univocal in supporting this hypothesis.

A prospective cohort study of 446,346 Taiwanese adults showed that active asthma is associated with adverse cardiovascular consequences (Strand et al., 2018). Atherosclerosis Risk in Communities (ARIC), a prospective study on the etiology of atherosclerotic-related diseases, demonstrated an association with increased carotid artery intima–media thickness (IMT) (Onufrak et al., 2007) and incidence of coronary heart disease (CHD) and stroke (Onufrak et al., 2008) in women with late-onset asthma, but not in those with child-onset asthma. Similar studies have also reached the conclusion that late-onset asthma inferred a greater risk of CVD (Lee et al., 2012; Tattersall et al., 2016). It was speculated that this may be due to the large overlap between the risk factors between late-onset asthma and CVD, few of which are linked to inflammation such as obesity, stress, estrogen-modulated inflammation, and increased numbers of eosinophils (Wenzel, 2012). Other studies confirmed that both asthma and allergy were independently associated with an increased risk of CHD (Iribarren, 2004; Kim et al., 2010; Iribarren et al., 2012), that asthma led to augmented vascular inflammation (Vijayakumar et al., 2013), and that patients with allergic rhinitis and asthma or chronic rhinosinusitis showed an increase of carotid IMT (Knoflach et al., 2005; Elcioglu et al., 2016). One study has shown there is a significant decrease in endothelium dependent vasodilatation in asthmatic patients (Yildiz et al., 2004), while other studies found they had significantly increased arterial stiffness (Augusto et al., 2017; Tuleta et al., 2017). Both are prognostic factors which are independent predictors of cardiovascular events. Interestingly, the latter study also included young asthmatic patients, suggesting that the biological mechanisms pertaining to cardiovascular comorbidity in asthma may also be present in childhood.

On the contrary, a large study investigating the association of self-reported, doctor diagnosed asthma and CVD in adults followed over 14 years, showed that asthma and duration of asthma did not associate with CHD (Schanen, 2005). Similarly, patients with allergic rhinitis had lower incidence of acute MI and cerebrovascular disease in a large retrospective, population-based, matched cohort study (Yoon et al., 2016); while another study investigating 61,899 Taiwanese patients affected by rhinitis and 123,798 age- and sex-matched controls, found they had decreased risk of developing acute ischemic stroke (Tseng et al., 2015). In summary, clinical and epidemiological data so far are unresolved.

A confounding factor to be considered may be the long-term use of asthmatic medication on the cardiovascular system. For example, one large cohort study demonstrated that only asthma patients using medications (particularly those on oral corticosteroids alone or in combination) were at a greater risk of developing CVD (Iribarren et al., 2012). On the contrary, a combination of inhaled corticosteroid and long-acting β2-agonist approved for the treatment of chronic obstructive pulmonary disease (COPD) and asthma led to improved lung function in subjects with COPD and comorbid CVD, without increasing CVD risk (Covelli et al., 2016). Consequently, further examination into the specific pathways which may be modulated by chronic use of anti-asthmatic drugs should be explored in relation to how they may contribute to the development of CVD.

A final consideration should be given to cigarette smoking, a major risk factor for CVD (GBD 2015 Risk Factors Collaborators, 2016). Compared with never-smokers with asthma, asthma patients who smoke have worse symptoms, increased chronic mucus hypersecretion, and more exacerbations, as well as an impaired therapeutic response to corticosteroids and a different inflammatory profile in sputum and blood (Thomson et al., 2013). Smokers with asthma have higher rates of cardiovascular comorbidities (Çolak et al., 2015). The endotype of smokers with asthma is more pro-inflammatory than the Th2 phenotype typically associated with atopic asthma (Peters et al., 2014; Fahy, 2015; Lambrecht et al., 2019). This pro-inflammatory phenotype may lead to increased CVD risk. Statins, which exert pleiotropic anti-inflammatory effects, reduced frequency of heart failure in the asthma–COPD overlap syndrome (Yeh et al., 2019), suggesting that a comparative study will be informative of communal mechanisms leading to exacerbated CVD and asthma in smokers.

In addition to controversial clinical observations linking asthma to atherosclerosis, there is also insufficient basic research in this area. One study which did explore this relationship yielded very interesting results when utilizing the ovalbumin (OVA)-alum sensitization/challenge model of allergic pulmonary inflammation in atherosclerotic apolipoprotein-E (apoE)^−/−^ mice. In fact, asthmatic apoE^−/−^ mice were found to have significantly larger and more vulnerable plaques compared to PBS-challenged apoE^−/−^ shams (Wang et al., 2014). This was attributed to elevated circulating Th2 and Th17 cells, along with their associated cytokines IL-4 and IL-17A respectively, at both early and advanced stages of pathology in the apoE^−/−^ mice with asthma. The group then assessed if suppressing the increased serum levels of IL-4 and IL-17A, using neutralizing antibodies, could attenuate disease progression in these mice. Remarkably, lesion size was reduced by inhibition of either IL-4 or IL-17A individually, but with an even greater effect observed when both were targeted in combination. Another study demonstrated that both chronic and acute allergic lung inflammation, induced in mice by ovalbumin sensitization and challenge, promoted atheroma formation, regardless whether lung inflammation occurred before, after, or at the same time as atherogenesis (Liu C.L. et al., 2016). Therefore these studies not only may support clinical data in finding that atherosclerosis pathology is exacerbated by asthma, but also proposes a potential method for clinical intervention, through targeting the proinflammatory cytokines which act at the interface of these two pathologies (Wang et al., 2014). However, it should be considered that the OVA-alum protocol, while useful for modeling asthmatic features and symptoms in mice, is likely not to be completely reflective of the complex and heterogeneous human disease (Aun et al., 2017). Consequently, further studies of this nature are required to fully elucidate the mechanisms and key players underpinning the potential accelerated development of atherosclerosis in asthma.

## Targeting Cytokines

As discussed previously, the CANTOS trial has demonstrated that targeting pro-inflammatory cytokines highly expressed in CVD can significantly improve clinical outcomes in patients (Ridker et al., 2017). Here we will discuss the cytokines of asthma which act to disrupt inflammatory homeostasis and consequently may exacerbate the progression of CVD.

The CANTOS trial utilized a monoclonal antibody to inhibit the multifunctional pro-inflammatory cytokine IL-1β, often referred to as the gatekeeper of inflammation due to its ability to regulate numerous immune cascades (Libby, 2017; Panahi et al., 2018). It is therefore unsurprising that IL-1β and some of its downstream targets have been implicated in asthma, particularly in the more severe forms of the disorder (Kim et al., 2017; Lambrecht et al., 2019).

IL-1β is activated by the caspase-1/NLRP3 inflammasome signaling which has been demonstrated to be causative in pathogenesis in two different OVA-mediated murine models of allergic pulmonary inflammation (Besnard et al., 2011; Kim et al., 2017). Deficiency in NLRP3 or IL-1R1 in mice which were subjected to airway sensitization and challenge of OVA significantly reduced cell infiltration and mucus production in the lung (Besnard et al., 2011). This model was performed without the use of aluminum adjuvant in order to better reflect naturally occurring disease, and the result determined to be due to a loss of the Th2 inflammatory allergic response (IL-5, IL-13, IL-33) which is classically associated with asthma. Similarly, Kim et al. utilized a model of severe steroid-resistant asthma (SSRA) and found that pharmacological inhibition of different members of the NLRP3 inflammasome signaling pathway suppressed the cardinal features of SSRA (Kim et al., 2017). In humans, both IL-1 receptor and NLRP3 inflammasome are elevated in the sputum of non-smoking adults with asthma (Simpson et al., 2014; Evans et al., 2018). These findings paired with the recent success of the CANTOS trial supports the notion that the NLRP3-IL-1β axis may be an appealing area for further investigation into a targetable link between asthma pathology and the development of CVD.

IL-33, another member of the IL-1 family, is found to be significantly increased in the serum of asthmatic patients (Momen et al., 2017; Ding et al., 2018). IL-33 functions as an “alarmin” which is released by the lung epithelial cells following stress or damage-induced necrosis, once released IL-33 promotes activation of the Th2-branch of the immune response (Robinson et al., 1992). Efficacy, safety, and tolerability of SAR440340 (anti-IL-33 mAb) is currently under investigation in patients with moderate-to-severe chronic obstructive pulmonary disease (COPD) (ClinicalTrials.gov Identifier: NCT03546907). However, the role of IL-33 in cardiovascular disease is controversial. While a few reports have shown that IL-33 induced endothelial cell activation and increased angiogenesis and vascular permeability, in contrast, others have demonstrated that treatment with IL-33 reduced experimental atherosclerosis (Miller et al., 2008; Choi et al., 2009; Aoki et al., 2010; Demyanets et al., 2011; Altara et al., 2018).

The predominance of the Th2 response in asthma has traditionally sparked controversy in relation to its association with atherosclerosis-related CVD, classically Th1-dominated pathologies. For example, IL-13, a prototypical Th2 cytokine, has been shown to protect from experimental atherosclerosis through the induction of alternatively activated macrophages (Cardilo-Reis et al., 2012). However, there is evidence that Th2 cytokines may also play a detrimental role in the CVD (Liu C. et al., 2016; Niccoli et al., 2018). Importantly, in the combined murine model described by Wang et al. expression and production of IL-4 was increased in the apoE^−/−^ mice after they were subject to allergic pulmonary inflammation, which was demonstrated to be partially causative in increasing plaque size and vulnerability (Wang et al., 2014). IL-4 is an important therapeutic target in asthma. The alpha subunit of the IL-4 receptor (dupilumab), that blocks both IL-4 and IL-13 pathways, has resulted in reduction of asthma morbidity, while the use of the anti-IL-4R failed in phase II (Eger and Bel, 2019). Mechanistically, IL-4 has been shown to increase endothelial permeability and dysfunction *via* cytoskeleton remodeling (Skaria et al., 2016). In addition, IL-4 can influence the activity of pro-inflammatory IL-6, resulting in exacerbation of the immune response *via* CD4+ T cell activation and inflammatory endothelium dysfunction (Rincón et al., 1997; Lee et al., 2010).

Upon exposure to several environmental antigens which trigger the Th2 response, IL-9 is also released and overexpression can lead to inflammation and airway hyperresponsiveness (Zhou et al., 2001). Elevated levels of IL-9 are observed in the sputum of asthmatic patients and expression in the nasal mucosa has been observed to increase in response to seasonal allergen changes (Zhou et al., 2001; Nouri-Aria et al., 2005; Lloyd and Hessel, 2010). However, the first randomized controlled trial to evaluate the effect of an anti-IL-9 monoclonal antibody showed no effect in adults with uncontrolled asthma (Oh et al., 2013). High levels of systemic IL-9 have been demonstrated to influence also atherosclerotic pathology, wherein administration of recombinant IL-9 in apoE^−/−^ mice enhanced inflammatory cell infiltration into lesions *via* modulation of VCAM-1 expression, resulting in enlarged plaques (Zhang et al., 2015).

Th1 and Th17- type responses have also been shown to play a role in asthma pathogenesis, and elevated levels of IFN-γ, TNF-α, and IL-17A can be found in the serum of asthmatic patients. This is of particular interest as Th1 and Th17 type responses are more characteristically associated with atherosclerosis (Sage and Mallat, 2017). It should be noted, however, that several of these mediators have also been shown to be produced by mast cells in asthma (Kennedy et al., 2013).

The pleiotropic IL-6 is expressed at high levels in asthma (Rincon and Irvin, 2012). Interestingly, IL-6 can translocate from inflamed lung to the systemic circulation in mice, and was also found to be elevated in the sputum of some patients with mild to moderate asthma (Neveu et al., 2010; Kido et al., 2011). These higher levels of IL-6 may contribute to driving CVD comorbidity in asthma. Interestingly, genetic variants which lead to higher circulating concentrations of IL-6 receptor (neutralizing IL-6 cell signaling) appear protective against CHD (Interleukin-6 Receptor Mendelian Randomisation Analysis (IL6R MR) Consortium et al., 2012). Interestingly, IL-6 trans-signaling is associated to risk of future cardiovascular events (Ziegler et al., 2019) and recently, epithelial IL-6 trans-signaling has been shown to define a new asthma phenotype with increased airway inflammation (Jevnikar et al., 2019). In experimental atherosclerosis, IL-6 mRNA expression was found in the atherosclerotic plaques of apoE^−/−^ mice (Sukovich et al., 1998). Further to this, the balance between IL-6 and anti-inflammatory IL-10 is believed to influence lipid homeostasis, plaque formation, and plaque morphology in mice (Schieffer et al., 2004). IL-6 is also well associated with abdominal aortic aneurysm (AAA) pathogenesis, primarily through modulating cell migration and infiltration (Nishihara et al., 2017). To date, however, IL-6 targeting has not been tested in secondary prevention of atherosclerosis and a phase 2a study, designed to evaluate the effects of sirukumab (human anti IL-6 monoclonal antibody) in subjects with severe poorly controlled asthma, was withdrawn (ClinicalTrials.gov Identifier: NCT02794519). In a recent small proof-of-concept clinical trial, a single dose of tocilizumab (IL-6 receptor blocker) was unable to prevent allergen-induced bronchoconstriction (Revez et al., 2019) (Australian New Zealand Clinical Trials Registry: ACTRN12614000123640).

Tumor necrosis factor-α (TNF-α), another cytokine implicated in both asthma and cardiovascular disease, at first appears to be a promising target for further research in targeting shared dysregulation in these two pathologies (Brightling et al., 2008; Welsh et al., 2017). Pharmacological inhibition of TNF-α activity with the monoclonal antibody infliximab, suppressed IL-4 production, adhesion molecule expression, and eosinophil infiltration in a mouse experimental model of allergic rhinitis, the pathology of which is similar to asthma (Mo et al., 2011). However, initial clinical trials performed to test this drug in asthma yielded underwhelming results, with no clinical benefits observed (Edwards and Polosa, 2007; Matera et al., 2010). On the contrary, etanercept, in a small cohort of 39 patients with severe corticosteroid refractory asthma, caused a small but significant improvement in asthma control (Morjaria et al., 2008) but using golimumab there was no favorable risk–benefit profile in patients with severe persistent asthma (Wenzel et al., 2009). No clear efficacy of anti-TNF treatments has been shown to date in CVD. One of the main limitations, is the fact that randomized controlled trials in chronic inflammatory diseases have been powered to demonstrate improvements in disease activity rather than in CVD endpoints (Welsh et al., 2017). However, meta-analysis of observational studies in patients affected by chronic inflammation suggest that those receiving TNF-α blockers are at lower risk of CVD (Roubille et al., 2015). More recently, the ENTRACE trial results demonstrated cardiovascular safety of tocilizumab (IL-6 receptor blocker) and etanercept (TNF-α blocker) in rheumatoid arthritis patients. Assessment of clinical efficacy, however, will require the enrolment of a much larger sample size (Giles et al., 2019).

In asthmatic hyperlipidemic apoE^−/−^ mice, T-regulatory cells (Tregs) were found to be decreased in the early stages of the pathology (Wang et al., 2014). Over the last decade, the anti-inflammatory cytokine IL-10, mainly produced by Tregs, has been under the spotlight in regards to its role in a variety of allergic diseases (Hawrylowicz, 2005). IL-10 is expressed on epithelial and endothelial cells of nasal mucosa in patients affected by allergic rhinitis and IL-10 serum levels are observed to be significantly lower in asthmatic patients compared to healthy controls (Muller et al., 2014; Raeiszadeh Jahromi et al., 2014). It is believed that enhancing IL-10 activity in allergic diseases such as asthma could be beneficial to pathology, and in CVD there is evidence that also supports this hypothesis. IL-10 deficiency significantly augments the development of atherosclerotic lesions in hyperlipidemic low-density lipoprotein receptor (LdlR)^−/−^ mice and also accelerates neointimal formation in hypercholesteremic ApoE*3-Liden mice (Potteaux et al., 2004; Eefting et al., 2007). Similarly, Tregs have been shown to be clearly protective in experimental atherosclerosis (Sage and Mallat, 2017), and two clinical trials are currently investigating the possibility to promote Treg expansion in patients with small abdominal aortic aneurysms (VIVAAA; ClinicalTrials.gov Identifier: NCT02846883), or with stable ischemic heart disease and acute coronary syndromes (LILACS; ClinicalTrials.gov Identifier: NCT03113773).

Another anti-inflammatory cytokine IL-37 expression was significantly reduced in the sputum of asthmatic children (Charrad et al., 2016), and was shown to be a key suppressor of asthma mediated mast cells (Conti et al., 2017) and airway inflammation and remodeling (Huang et al., 2018; Meng et al., 2019). IL-37 has also been found to exert athero-protective effects *via* the modulation of dendritic cell maturation, macrophage activation and by inducing Treg responses (Chai et al., 2015; Ji et al., 2017; McCurdy et al., 2017; Liu et al., 2018). Enhancing the activity of anti-inflammatory cytokines such as IL-10 and IL-37, which have protective roles in both disease states could be another potential avenue for pharmacological intervention for asthmatic patients who develop or are at high risk for developing CVD as a comorbidity.

## Conclusion

To date the clinical evidence supporting the hypothesis that asthma confers high risk of various cardiovascular pathologies to patients is not univocal, and the specific biological mechanisms which may facilitate the development of cardiovascular comorbidity remain to be fully determined for potential pharmacological intervention to be explored. As inflammation plays a pivotal role in both atherosclerosis and asthma, potential targets for this research are cytokines whose dysregulation have notable effects in both diseases.

## Author Contributions

DG and LM equally contributed to the writing of the manuscript. CM, TG, and PM revised and finalized the text. All authors have read and approved the final manuscript.

## Funding

Our work is supported by the British Heart Foundation (BHF) grants PG/12/81/29897, RE/13/5/30177, FS/16/55/32731, and PG/19/84/34771; the Engineering and Physical Sciences Research Council (*EPSRC*) grant EP/L014165/1; the European Commission Marie Skłodowska-Curie Individual Fellowships 661369 and the European Research Council (Project Identifier: 726318).

## Conflict of Interest

The authors declare that the research was conducted in the absence of any commercial or financial relationships that could be construed as a potential conflict of interest.
